# Supplementary material for: The interactome of intact mitochondria by cross-linking mass spectrometry provides evidence for coexisting respiratory supercomplexes
Source: Mol Cell Proteomics. 2017 Dec 8;17(2):216–32. doi: 10.1074/mcp.RA117.000470 (PMC5795388; doi:10.1074/mcp.RA117.000470)
Supplement: Supplemental Data [file supp_RA117.000470_133922_0_supp_23978_fzffwf.zip › spectra_annotation/mito_spectra_annotation/409-1-9-1-10-1.pdf]

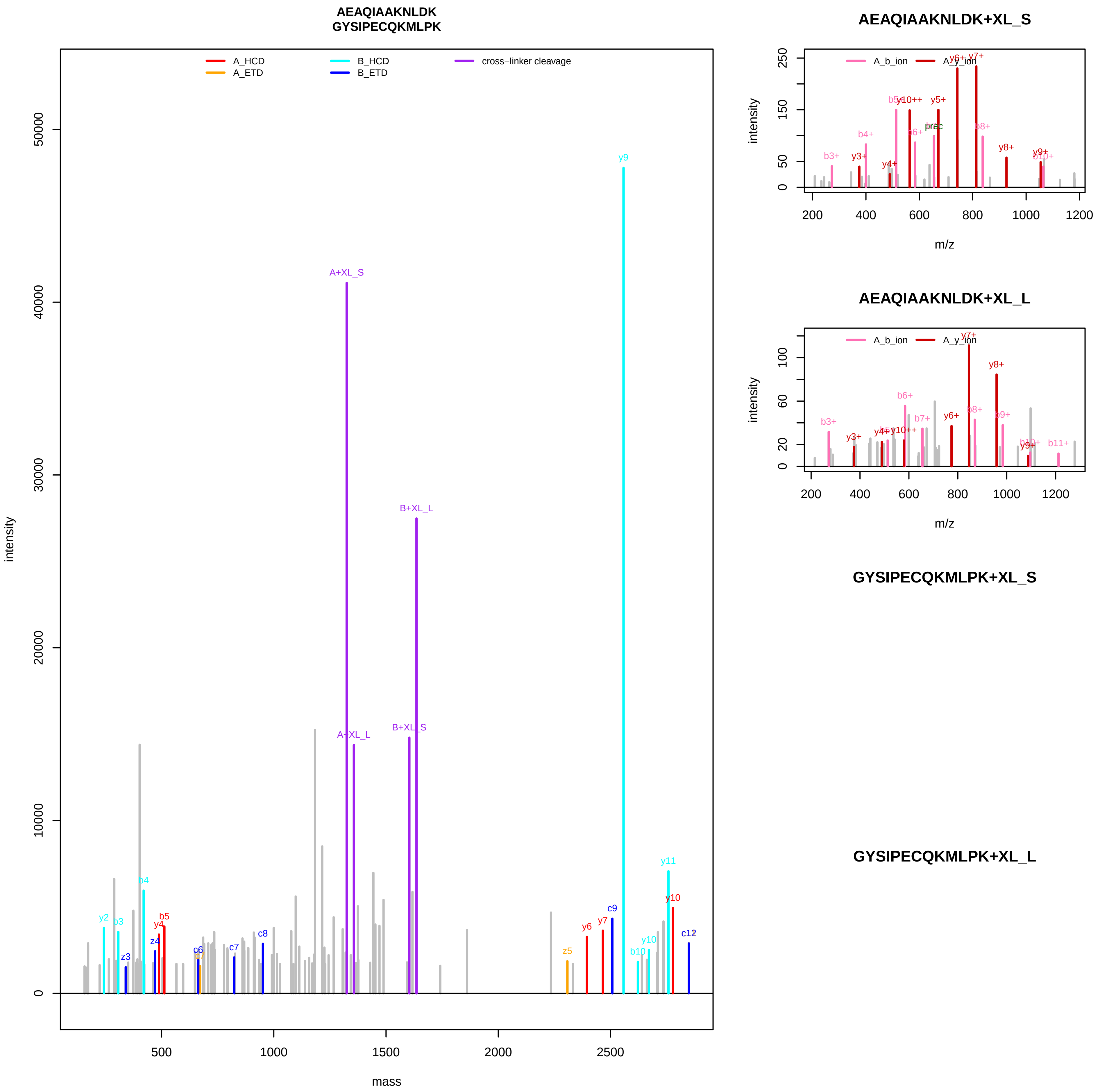
AEAQIAAKNLDK
GYSIPECQKMLPK
A_HCD
A_ETD
B_HCD
B_ETD
cross−linker cleavage
intensity
mass
0
10000
20000
30000
40000
50000
500
1000
1500
2000
2500
y9
A+XL_S
B+XL_L
A+XL_L
B+XL_S
y2
b3
z3
b4
z4
y4
b5
c6
c7
c8
z5
y6
y7
c9
b10
y10
y11
y10
c12
AEAQIAAKNLDK+XL_S
A_b_ion
A_y_ion
intensity
m/z
0
50
150
250
200
400
600
800
1000
1200
b3+
y3+
b4+
y4+
b5+
y10++
b6+
prec
y5+
y6+
y7+
b8+
y8+
y9+
b10+
AEAQIAAKNLDK+XL_L
A_b_ion
A_y_ion
intensity
m/z
0
20
60
100
200
400
600
800
1000
1200
b3+
y3+
y4+
y10++
b6+
b7+
y6+
y7+
b8+
y8+
b9+
b10+
y9+
b11+
GYSIPECQKMLPK+XL_S
GYSIPECQKMLPK+XL_L
